# A randomized controlled clinical study of acupuncture therapy for Seborrheic alopecia in young and middle ages: Study protocol clinical trial (SPIRIT compliant)

**DOI:** 10.1097/MD.0000000000019842

**Published:** 2020-04-24

**Authors:** Qiulian Chen, Yun Tao, Longjun Wang, Jinfang Zhang, Bichen Sun, Xiaoli Yang

**Affiliations:** a Graduate student of HeBei North University; b Department of Dermatology,Huabei Petroleum General Hospital; c Graduate Student of Hebei University of Chinese medicine; d Department of Dermatology, The First Hospital of Hebei Medical University; e Department of Traditional Chinese Medicine, Huabei Petroleum General Hospital; f Qin Huang Dao Hospital of Traditional Chinese Medicine of HeBei North University,China.

**Keywords:** acupuncture, protocol, randomized controlled trial, Seborrheic alopecia

## Abstract

**Introduction:**

Seborrheic alopecia (SA) has clinical manifestations, duration of disease, and priorities. In the current situation where there are many and complicated clinical treatments, Western medicine treatment can delay and control the development of the disease and promote hair regeneration. However, some patients may aggravate symptoms after taking the drug, and the condition is easy to repeat after stopping the drug. Acupuncture is an important method for non-surgical treatment of SA, and it has various methods, low side effects, high safety, and simple and economical. Therefore, we will use a clinical randomized controlled study to explore the effect of acupuncture on SA, and provide a new idea and reference for the treatment of this disease.

**Methods/design:**

We will select 60 patients diagnosed with SA. They will be randomly divided into intervention group and control groups. The control group will be given conventional treatment measures. The intervention group will receive acupuncture. Efficacy will be evaluated by comparing the skin lesion score and dermatological quality of life index before and after treatment.

**Discussion:**

This trial may provide evidence regarding the clinical effectiveness, safety, and cost-effectiveness of acupuncture for patients with SA.

**Trial registration number:**

CTR2000030430

## 1 Introduction

Seborrhoeic alopecia (SA) is also called androgenic alopecia. SA is an androgen-dependent polygenic hereditary hair loss disease. The clinical manifestations of SA are mostly excessive secretion of scalp oil, greasy hair, large formation of dandruff, and sometimes itchy scalp.^[1]^ SA is more common in young men, and hair loss is often on the sides of the forehead and the top of the head. The number of women with SA is also increasing, but the symptoms are relatively mild.^[2]^ Because the disease affects aesthetics, it brings great mental stress and psychological burden to patients.^[3]^ The pathological characteristics of SA are mainly reflected in the following changes: changes in hair growth cycle, changes in subcutaneous blood flow, changes in sebaceous glands, and changes in hair follicle stem cells.^[4]^ The hair growth of SA patients showed changes in both the growth period and the stationary period. The proportion of hair growth in SA patients are shortened, the growth period increased, and the proportion of growth period and resting hair follicles decreased. The mechanism that causes these pathological changes is not completely clear. Recent studies have shown that the occurrence of SA is related to heredity, serum androgen levels, local androgen receptors, scalp subcutaneous blood flow, endocrine function, and psychosocial factors. About the treatment of this disease, oral medication is currently used.^[5]^ The most commonly used oral drug in clinical practice is finasteride. Clinical studies have shown that its effect in treating hair loss is clear, but the side effects of erectile dysfunction caused by reduced levels of sex hormones caused by long-term use are inevitable. In terms of topical use, minoxidil is mostly used clinically. Its mechanism may be to activate potassium channels, expand the subcutaneous blood vessels, and regenerate the hair and papilla blood vessels of the head.^[6]^ However, to obtain long-term effects as a topical drug, continuous medication is required.

SA has a relatively complete understanding in traditional Chinese medicine (TCM) theory. From ancient times to now, with the gradual improvement and development of TCM theory, the understanding of hair loss has become more detailed and a more complete diagnosis and treatment system has been formed. Since modern times, TCM has developed rapidly and has a deeper and deeper understanding of disease. Combined with the clinical symptoms of hair loss in modern medicine, hair loss is divided into seborrheic hair loss and alopecia areata. The former is defined as progressive loss of hair on the top of the head, which may be accompanied by strong oil secretion, itching, and increased dandruff; the latter is manifested as a sudden flaking of hair on the head, with no other accompanying symptoms.^[7]^ SA clinical manifestations, duration of disease, priorities vary. In the current situation of multiple and complicated clinical treatments, Western medicine oral medication can delay and control the development of the disease and promote hair regeneration.^[8]^ However, some patients may aggravate symptoms after taking the drug, and it is very easy to relapse after stopping the drug. As an important part of TCM, acupuncture has been widely used in the treatment of SA in recent years. By dialectical treatment, acupuncture points can be taken along with the evidence, which can play a role in replenishing Qi and regenerating essence, and nourishing liver and kidney. Acupuncture can improve local microcirculation, make the body full of Qi and blood, and then maintain hair. Acupuncture treatment of SA is not only a specimen that is effective, but also has small toxic and side effects, low price, and is easy to popularize. Therefore, we plan to use randomized controlled research methods to explore the clinical efficacy of acupuncture in the treatment of SA, and provide a way of thinking and reference for the treatment of SA.

## 2 Methods/design

### 2.1 Study design and settings

**Figure 1:**
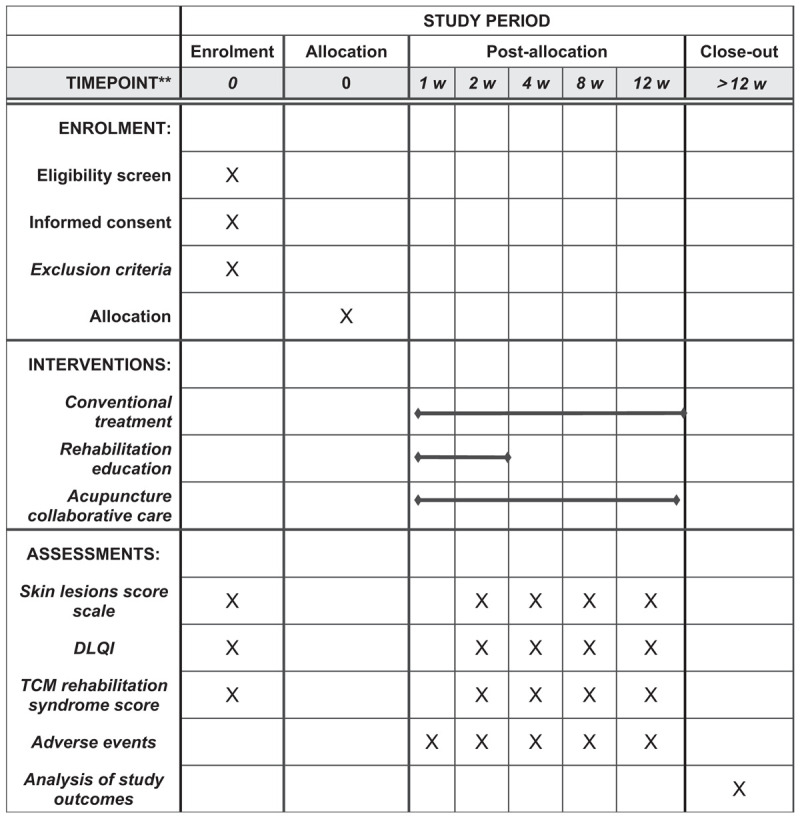
SPIRIT figure for the schedule of enrollment, interventions, and assessments. DLQI = dermatology life questionnaire index, SPIRIT = standard protocol items: recommendations for interventional trials, TCM =traditional Chinese medicine.

The study will be a randomized controlled trial divided into 2 parallel groups. This protocol is based on the Standard Protocol Project: Recommended Guidelines for Interventional Trials. If they agree, they will sign an informed consent form. Following the schedule described in Figure 1, only participants who read and agreed to the agreement and signed informed consent will be allowed to participate in the study. After patients will be included in this clinical observation, the research guidelines of randomized controlled trials will be strictly followed, and the randomized number table method are going to be used to divide the admitted patients into test groups and control groups on average.

### 2.2 Participants

**Figure 2:**
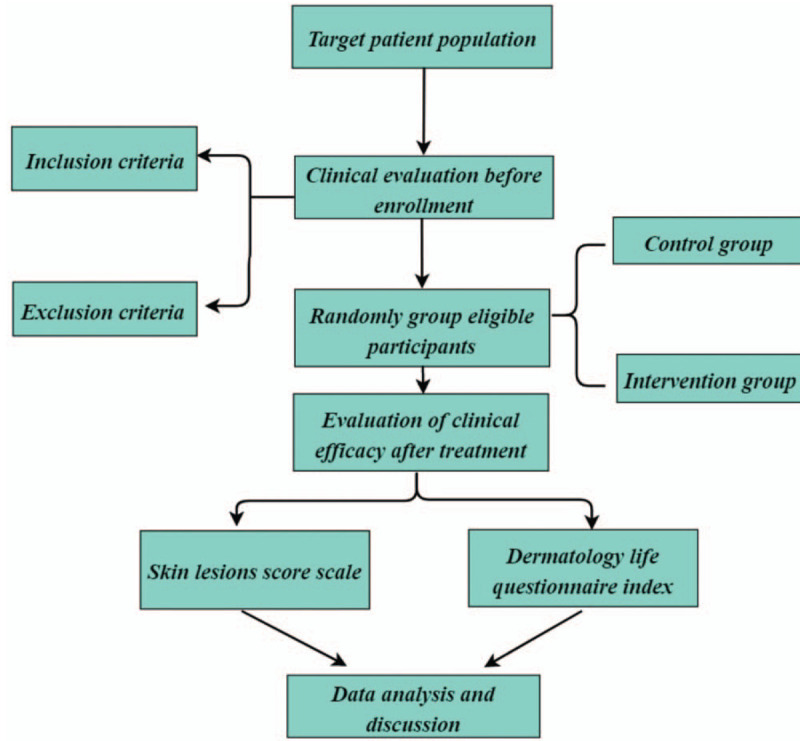
Study design flow chart.

All cases in this study will come from acupuncture clinics and ward and dermatology clinics. This study intends to include a total of 60 samples. Eligible cases were randomly divided into treatment group and intervention group according to the digital randomization grouping method. (Fig. 2).

#### 2.2.1 Diagnostic criteria

The diagnostic criteria for Western medicine SA will refer to “*Clinical Dermatology”* (Edited by Zhao Yan, Jiangsu Science and Technology Press, December 2009, 1st edition). The proposed criteria are as follows:

(1) The clinical manifestations of men start from both sides of the forehead, and the hair gradually becomes thinner and thinner and extends toward the top of the head. The forehead hairline receded backward; the forehead was raised, forming an “M” -shaped high amount.
(2) The clinical manifestations of women are that the hair on the top of the head is gradually thinner, the hairline on the forehead is retained, and hair remains on the pillow.
(3) The skin lesion area is smooth as a mirror, and slender hairs are visible.
(4) No obvious conscious symptoms or itching.

The diagnostic criteria for TCM SA will refer to the relevant content in the “Guiding Principles for Clinical Research of New Chinese Medicine”. The main symptoms: shiny hair, greasy dandruff, often a few hair sticks, hair thinning, and bald. Secondary symptom: accompanied by appetite, stool. Red tongue, yellow greasy fur, and pulsating veins.

#### 2.2.2 Inclusion criteria

(1) 18 to 50 years old (young and middle-aged), no gender restriction;
(2) Western medical diagnostic standards that meet SA;
(3) No other drugs that have an impact on the results of this study within 1 month before enrollment;
(4) Sign the informed consent.

#### 2.2.3 Exclusion criteria

Patients will be excluded if they meet the following criteria:

(1) Those who are younger than 18 years old or older than 50 years;
(2) Other types of hair loss such as alopecia areata, congenital or traumatic hair loss, and chemotherapy or postpartum alopecia.
(3) Patients with mental illness.
(4) Those with local skin inflammation, erosion, or exudation.
(5) Patients with previous hair transplantation.
(6) Those with more serious underlying diseases.
(7) Those who are pregnant or lactating.
(8) Patients known to be allergic to the drugs contained in the test.

Note: Those who meet any of the above criteria are not allowed to join this study.

#### 2.2.4 Case rejection or shedding criteria

(1) Participants found not to meet the inclusion criteria after inclusion;
(2) In the course of treatment, patients who used the banned drugs specified in the plan themselves were used;
(3) Those who cannot follow the doctor's orders due to reasons other than side effects, who have caused obstacles in determining the efficacy and safety assessment;
(4) Those who were lost to follow-up during treatment and patients who requested to withdraw from the trial due to their own reasons.

#### 2.2.5 Suspension criteria

(1) In the treatment of this study, the patient was unexpectedly pregnant or had serious adverse reactions, and it was difficult to continue to receive acupuncture treatment.
(2) During the implementation of this study, there was an obvious exacerbation or complications.

### 2.3 Interventions

#### 2.3.1 Control group

Patients in the control group will receive regular shampoo care. The specific content is: keep washing your hair with warm water once every 2 days to reduce seborrhea and scalp itching to reduce scalp and hair damage caused by scalp pain and itching. Shampooing should not be too frequent. Frequent shampooing will promote the secretion of sebaceous glands and increase seborrhea. The intervention group and the control group were treated 3 times a week, 4 weeks as a course of treatment, and a total of 3 courses of treatment.

#### 2.3.2 Intervention group

The intervention group will be treated with acupuncture on the basis of the control group. We will select *Baihui*, *Fengchi*, *Sishencong, Zusanli, Neiting*, and *Taichong* as the main points. Secondary acupuncture points: those with hair loss on both sides of the crotch, plus *Touwei*; those with scalp pruritus, enlarged *Dazhui*; those with more oil secretion, plus *Shangxing*. Specific operation method: the patient takes the prone position, the forehead rests on the square pillow; conventional disinfection, after positioning the needle, hold the acupuncture needle on the left and place it on the acupoint; use the index finger to quickly tap the exposed needle handle to make the needle tip stab under the skin; then remove the needle tube and pierce the needle into the acupoint. Insert the needle perpendicular to the skin surface with a 1.5 inch needle. After leaving it for 30 minutes, the needle was started. The positioning and operation of common acupuncture points are performed according to the positioning and operation of acupuncture points in the second edition of the Chinese “Twelfth Five-Year Plan” textbook “Meridian Acupoints” (China Traditional Chinese Medicine Press, chief editor Shen Xueyong). Needle selection: Sterile acupuncture needles (0.25 × 25 mm, 0.30 × 40 mm) produced by Suzhou Acupuncture Supplies Co. Ltd.

#### 2.3.3 Basic treatment

For the sake of patient's health, this study will provide basic treatment-rehabilitation education for 2 groups of patients.

(1) SA patients should pay attention to the combination of work and rest, especially mental workers should pay attention to avoid long-term tension and excessive brain use. Consistent exercise is the key to strengthening your body and protecting your hair. Regular exercise is also one of the ways to eliminate tension, and tension is also an important cause of hair disorders.
(2) Diet adjustment needs to pay attention to the balance of nutritional intake, the richness of food types, and the reasonable intake of various foods to ensure the necessary nutrients for hair growth. In particular, it is necessary to ensure the intake of nutrients that are beneficial to hair growth, such as soybeans, walnuts, black sesame, and cereals that are rich in vitamins and trace elements; reduce fat intake, especially animal fats and sugars, and spicy Food intake does not aggravate sebum spillage and affect hair growth.
(3) During the treatment, we will briefly explain the cause of SA to patients, and inform the prognosis of the disease and daily precautions.

### 2.4 Outcome measures

#### 2.4.1 Primary outcome measures

Primary outcome measures will be assessed using symptom scores. The clinical efficacy will be determined by referring to the “Guiding Principles for Clinical Research of New Chinese Medicines” and “Clinical Dermatology”. Establish a standard for determining the efficacy (refer to the 4-point scoring method for clinical research guidelines for new Chinese medicines) to determine the efficacy index using the nimodipine method. That is [(pre-treatment points-post-treatment points) ÷ pre-treatment points] × 100%. Clinical total effective rate = clinical cure rate + clinical apparent efficiency + clinical effective rate. In addition, we will refer to the relevant standards in “Clinical Dermatology” and develop clinical efficacy observation scores based on clinical practice. Hair loss (comb test), scalp condition (greasy scalp, itching, dandruff, folliculitis), hair regeneration (new hair density, new hair texture) total 7 indicators, divided into 4 levels for evaluation.

#### 2.4.2 Secondary outcome measures

Secondary outcome measures will be assessed based on the psychological status of SA patients. We will use the Dermatology Life Questionnaire Index and hair growth self-evaluation questionnaire to evaluate specific indicators.

### 2.5 Randomization and blinding

According to the related literature and the actual situation, 60 cases were collected. Sixty cases will be referred to the random grouping method in “Chinese and Western Medicine Clinical Research Methodology” published by Science Press in 2008. The patients will be randomly divided into 30 cases in the treatment group and 30 cases in the control group. Grouping method: The computer generates random numbers, makes cards into envelopes, and draws them in the order of patient visits. Patients corresponding to the last digits are assigned to the treatment group, and patients corresponding to the last digits are assigned to the control group.

### 2.6 Statistical analysis

SPSS for windows 24.0 statistical analysis software will be used for calculation, and normality test and homogeneity test of variance will be performed on each group of data. For measurement data in which the data conforms to the normal distribution, we will use the mean ± standard deviation. For non-normally distributed measurement data, the median ± quartile interval is used. General data comparison between the 2 groups using independent sample *t* test. Comparisons before and after treatment will be performed using *t* test for paired data. One-way analysis of variance will be used for comparison between groups. X^2^ test will be used for count data, and nonparametric rank sum test will be used for rank data. All statistical tests are two-sided. *P* < .05 indicates a significant difference.

### 2.7 Data management

Information obtained from the evaluation of each participant will be recorded on a paper print-out. The information will then be handwritten on a paper document case report form and entered into an Excel file for future statistical analyses. In accordance with the Personal Information Protection Act, the names of all participants will not be disclosed, and a unique identifier number given during the trial will be used to identify participants. All of the participants will be informed that the clinical data obtained in the trial will be stored in a computer and will be handled with confidentiality. The participants’ written consent will be stored by the principal investigator.

### 2.8 Ethics

The study protocol is going to be approved by the ethics committee of the Huabei Petroleum General Hospital. The study will be conducted under the Declaration of Helsinki principles, as well as following the norms of good clinical practice. Recruitment of patients has not started in this study. The study plan will be submitted to the ethics committee of the Huabei Petroleum General Hospital for review. We will not start recruiting participants without the consent of the ethics committee. The protocol of this study has been registered in the Chinese Clinical Trial Registry with the number ChiCTR2000030430.

## 3 Discussion

Modern research has shown that cerebral cortical function is mapped to corresponding parts of the scalp.^[9,10]^ Acupuncture stimulates the function of the cerebral cortex to locate the corresponding scalp area, improves brain electrical activity, and increases the alpha wave index voltage. The alpha wave can relax one's spirit, thereby achieving the effect of regulating emotions.^[11]^ Other studies have shown that acupuncture of the head can cause changes in cerebral blood flow. Hemorheology showed a marked improvement in cell aggregation and a decrease in blood viscosity.^[12]^ The cerebral blood flow chart shows that the average amplitude is high, the inflow time is shortened, and the changes in left and right cerebral blood flow tend to be balanced.^[13]^ Therefore, acupuncture can relax blood vessels, improve blood vessel elasticity, and increase cerebral blood flow. Modern medical research has found that SA is related to the accumulation of dihydrotestosterone in the hair follicle.^[14]^ Acupuncture on the head can regulate the local blood flow of the head, can antagonize the accumulation of dihydrotestosterone, and achieve the effect of treating SA.^[15]^ In addition, SA brings stress to patients and may produce negative emotions such as anxiety and depression. Acupuncture on the head improves brain electrical activity and enhanced alpha waves can relax one's spirit and avoid a series of negative effects such as anxiety and depression.

An important feature of hair growth is its periodicity. The reason is that the growth of hair follicles is periodic.^[16]^ The existence of hair follicles is a prerequisite to ensure hair growth and replacement. The growth cycle is divided into 3 phases: growing phase, degenerative phase and resting phase. About 90% to 95% of normal hair follicles are in the growth phase, 1% enter the degenerative phase, and 5% to 10% are the resting phase.^[17,18]^ When the resting phase is reached, the hair falls off, and the hair follicles enter the next growth cycle. The duration of the growth phase determines the length of the hair. The first long-term occurrence is about 2 to 6 years, the regression period is 2 to 3 weeks, and the rest period is 2 to 3 months.^[19]^ The proportion of hair follicles during growth and resting determines the degree of hair thinning. When the growth period is shortened and the number of hair follicles is increased during the resting phase, clinical signs of exfoliation occur.^[20]^ There are many clinical studies on SA, but the overall study design is not rigorous. Some clinical reports did not establish a control group or did not follow random and blind methods. Efficacy evaluations in most studies are highly subjective. Such as hair growth, itching degree, amount of dandruff, etc, lack of objective quantitative standards, evaluation standards are not unified and objective. Therefore, we will use a standardized clinical randomized controlled research method to evaluate the efficacy of acupuncture treatment of SA. The intervention group and the control group were treated 3 times a week, with 4 weeks as a course of treatment and a total of 3 courses of treatment. This is beneficial to reducing the effect of the hair rest cycle on the experimental results. We hope to provide a higher level of evidence for acupuncture treatment of SA, while also providing patients with more treatment options.

## Acknowledgments

The authors would like to thank all the trial participants. The authors are grateful for the support for this study: trial coordinating team, surgical staff, nurses, and research departments.

## Author contributions

QLC, YT and LJW designed the study protocol and drafted the manuscript. JFZ reviewed the study protocol and drafted the manuscript. BS is responsible for the statistical design and analysis as trial statistician. All authors carefully read and approved the final version of the manuscript. XLY participated in the design and coordination of the study. All authors read and approved the final manuscript.
